# Bacterioruberin-Rich Carotenoid Extract Protects Human PBMCs Against UV-Induced Oxidative Stress and Apoptosis: Insights into Mitochondrial and Cell Survival Mechanisms

**DOI:** 10.3390/md24100335

**Published:** 2026-09-26

**Authors:** Miguel Medina-García, Pascual Martínez-Peinado, Alicia Navarro-Sempere, Yolanda Segovia, Sandra Pascual-García, Andrés Baeza-Morales, Carolina Pujalte-Satorre, Raúl Cobo, Magdalena García, Micaela Giani, Rosa María Martínez-Espinosa, José Miguel Sempere-Ortells

**Affiliations:** 1 Immunology, Cellular and Developmental Biology Group, Department of Biotechnology, University of Alicante, Ap. 99, E-03080 Alicante, Spain; miguel.medina@ua.es (M.M.-G.); alicia.navarro@ua.es (A.N.-S.); yolanda.segovia@ua.es (Y.S.); sandra.pascual@ua.es (S.P.-G.); andres.baeza@ua.es (A.B.-M.); cps58@alu.ua.es (C.P.-S.); raul.cobo@ua.es (R.C.); m.garcia@ua.es (M.G.); josemiguel@ua.es (J.M.S.-O.); 2 Biochemistry and Molecular Biology and Edaphology and Agricultural Chemistry Department, Faculty of Sciences, University of Alicante, Ap. 99, E-03080 Alicante, Spain; micaela.giani@ua.es (M.G.); rosa.martinez@ua.es (R.M.M.-E.); 3 Applied Biochemistry Research Group, Multidisciplinary Institute for Environmental Studies “Ramón Margalef”, University of Alicante, Ap. 99, E-03080 Alicante, Spain

**Keywords:** ultraviolet (UV), apoptosis, carotenoids, antioxidant, bacterioruberin, PBMCs

## Abstract

Ultraviolet (UV) radiation is a major environmental stressor that disrupts cellular integrity by inducing oxidative stress, impairing mitochondrial function, and altering immune cell activity, ultimately compromising cellular homeostasis. In this context, carotenoids are widely recognized for their antioxidant and immunomodulatory properties, with C_50_ carotenoids emerging as a promising subgroup with enhanced biological activity compared with classical C_40_ carotenoids. Among them, bacterioruberin (BR), a red-pigmented C_50_ carotenoid mainly produced by haloarchaea, has attracted increasing interest due to its strong antioxidant and cytoprotective potential. Accordingly, this study investigated the mechanisms underlying the protective effects of a bacterioruberin-rich carotenoid extract (BRCE) against UV-induced damage in human peripheral blood mononuclear cells (PBMCs). PBMCs were pre-incubated with BRCE prior to UV exposure and intracellular reactive oxygen species (ROS), mitochondrial membrane potential (Δψm), caspase-3 activation, and the expression of B cell lymphoma 2 (Bcl-2) and granzyme A were evaluated using fluorescence-based assays via confocal microscopy and flow cytometry. BRCE attenuated oxidative stress, preserved mitochondrial membrane potential, reduced caspase-3 activation, and modulated Bcl-2 and granzyme A expression, with more pronounced effects observed at low concentrations. Overall, BRCE exerted a coordinated cytoprotective effect through modulation of oxidative stress, mitochondrial integrity, and apoptosis-related signaling pathways. These findings provide novel insight into the cellular effects associated with the bioactivity of bacterioruberin and support the growing interest in C_50_ carotenoids as promising photoprotective and immunomodulatory compounds.

## 1. Introduction

Ultraviolet (UV) radiation is a major environmental stressor that affects cellular and immune function [1]. Chronic or excessive UV exposure has been associated with disrupted redox homeostasis, the induction of inflammatory responses, and impaired immune cell function, ultimately contributing to tissue damage and immune dysregulation [2,3]. Given its widespread environmental exposure and its detrimental biological effects, understanding the cellular mechanisms involved in UV-induced damage remains an important area of research. Ultraviolet B (UVB; 280–320 nm) radiation is particularly well known to inflict direct DNA damage, provoke reactive oxygen species (ROS) production, disrupt mitochondrial function, and ultimately trigger apoptosis in human cells [4]. In immune cells, such as peripheral blood mononuclear cells (PBMCs), UVB exposure has been associated with oxidative stress and activation of apoptotic pathways, contributing to immunosuppression and compromised cellular defenses [5,6]. Apoptosis triggered by UVB is often mediated through ROS-dependent mitochondrial dysfunction, leading to loss of mitochondrial membrane potential, cytochrome c release, and activation of caspase-9 and caspase-3, together with modulation of B cell lymphoma 2 (Bcl-2) family proteins [7]. In addition, redox-sensitive signaling pathways such as Nrf2 may be impaired under UVB exposure, weakening endogenous antioxidant defenses [8]. Overall, the balance between oxidative stress, mitochondrial integrity, and apoptotic signaling determines whether cells undergo survival or programmed cell death. Given the central role of oxidative stress in UV-induced cellular damage, considerable efforts have been directed toward the identification of bioactive compounds with the potential to enhance cellular resilience against UV-induced stress.

In recent years, natural antioxidants have attracted considerable attention as potential modulators of oxidative stress and immune function. Among them, carotenoids constitute a large family of naturally occurring pigments widely distributed in plants, algae, and microorganisms, characterized by their strong antioxidant capacity and their ability to quench reactive oxygen species generated under diverse stress conditions [9]. Accordingly, carotenoids have been extensively investigated for their biological properties, particularly their antioxidant and immunomodulatory activities, which are closely associated with the regulation of oxidative stress, inflammation, and immune cell function [10]. This interest has also expanded to their role in mitigating UV-induced cellular damage. Several studies have demonstrated that carotenoids can reduce oxidative injury and attenuate the harmful effects of UV radiation in different tissues [9,11]. Consistent with these observations, recent clinical findings have shown that dietary carotenoid supplementation can protect human skin against UV-induced damage, highlighting the translational potential of carotenoid-based interventions for the prevention of photoaging and other UV-associated alterations [12]. These findings support the continued search for novel carotenoids with enhanced biological activity and improved protective properties.

Among emerging carotenoids, those containing C_50_ carbon chains have recently attracted considerable scientific attention due to their enhanced biological activities compared with conventional C_40_ carotenoids [13]. Within this group, bacterioruberin (BR), a red-pigmented carotenoid mainly produced by haloarchaea, has emerged as one of the most promising candidates [14]. The molecular structure of BR, characterized by an extended conjugated double-bond system and additional hydroxyl groups, has been previously reported to confer enhanced antioxidant performance compared with classical C_40_ carotenoids [15]. A few recent papers have described the role of BR in the cellular structure/dynamics of the microorganisms able to produce it, concluding that BR exerts a key role as an antioxidant to protect the cells against high ionic strength, high sun radiation, and water scarcity (main stressing features that characterized the natural environments occupied by haloarchaea as major microbial populations) [16]. This prominent role as an antioxidant under stressful conditions (which are also characteristic of human cells affected by various pathological processes) alerted the scientific community to the potential role BR could play as an antioxidant and/or immunomodulatory compound. Consequently, BR has been investigated for a wide range of biological activities, including antioxidant, antiglycemic, antilipidemic, and immunomodulatory effects [17,18,19,20,21]. In addition, BR has demonstrated selective antiproliferative activity in several tumor cell models, highlighting its context-dependent biological effects and potential therapeutic value [22]. Collectively, these findings position bacterioruberin as an emerging C_50_ carotenoid with growing scientific and biomedical relevance, particularly in the context of oxidative stress-related cellular responses. In line with this, our previous work showed that a bacterioruberin-rich carotenoid extract (BRCE), at low concentrations, significantly reduced UVB-induced apoptosis and improved cell viability in PBMCs [23]. However, although these findings established the cytoprotective potential of BRCE under UVB-induced stress, the molecular mechanisms responsible for these effects remain poorly understood.

Therefore, the present study was designed to further characterize the protective effects of BRCE in human PBMCs exposed to UVB irradiation and to elucidate the cellular mechanisms underlying its bioactivity. Despite previous evidence supporting its cytoprotective effects, the molecular basis of BRCE-mediated protection remains unclear. A better understanding of these mechanisms will not only advance current knowledge of bacterioruberin bioactivity but also provide valuable insight into the potential of C_50_ carotenoids as novel candidates for protecting immune cells against UV-induced oxidative and cellular damage.

## 2. Results

### 2.1. Intracellular ROS Measurement

With respect to the results obtained from the ROS analysis, differences were observed between the experimental conditions (Figure 1). In samples that were not exposed to UV light (blue bars), fluorescence intensity remained relatively low and stable across the different doses of BRCE, with no marked variations compared to the control condition. However, at the highest dose (75 µg/mL), we observed a reduction compared to the control without BRCE. In contrast, in samples exposed to UV radiation (purple bars), a higher fluorescence intensity was observed overall. Within these UV-treated samples and in the absence of BRCE, exposure to UV light results in an increase in intracellular ROS levels. However, increasing the BRCE dose led to a decrease in fluorescence intensity, reaching statistical differences at the 2.7 µg/mL dose. This effect was even more marked at higher doses of BRCE, where a pronounced and abrupt decrease in ROS levels was observed.

### 2.2. Analysis of Mitochondrial Membrane Potential (Δψm)

First, Δψm was assessed by means of fluorescence microscopy using Rhodamine 123, enabling qualitative visualization of mitochondrial polarization changes under the different experimental conditions (Figure 2A). Panels show representative images of cells from one donor with and without UV exposure. In the first row of non-UV-exposed cell images, fluorescence intensity remains relatively constant up to 25 µg/mL, where a slight decrease in signal becomes apparent. In contrast, the second row, which corresponds to the same BRCE concentrations but following UV exposure, shows a very low fluorescence intensity in the control sample without BRCE. However, an increase in fluorescence intensity is observed in 1 µg/mL and 2.7 µg/mL, reaching higher levels compared with the UV-only control. In the sample of 8.5 µg/mL, a slight increase is still evident, whereas at higher concentrations, the signal progressively decreases again, similar to what was observed in the control without BRCE.

In addition to the qualitative observation of the microscopy images, the mean grey values were also measured using ImageJ to quantify the differences observed by means of fluorescence microscopy. This analysis allowed the fluorescence signal of the cells in each condition to be quantified, providing a numerical comparison between the control samples and those incubated with the different BRCE doses, both in the presence and absence of UV irradiation (Figure 2B). Fluorescence intensity was quantified by measuring mean gray values in representative cells from each condition per donor. Background fluorescence was subtracted from all images prior to analysis. For each condition, the same region of interest (ROI) and acquisition settings were used across all samples to ensure comparability. Multiple fields of view were analyzed per donor, and values were averaged to obtain a representative measurement for each individual prior to statistical analysis. In samples incubated with the different doses of BRCE without UV, no differences were observed between the control without BRCE and the lower doses (between 1 µg/mL and 8.5 µg/mL). However, as BRCE concentration increased (25 µg/mL and 75 µg/mL), a progressive decrease in fluorescence intensity was observed compared with the control group, reaching statistical significance. In contrast, when analyzing the samples that were exposed to UV light, a significant increase in fluorescence intensity was observed at the lower BRCE doses (1 µg/mL and 2.7 µg/mL) compared with the control condition. Also in this case, as the BRCE dose increased, a reduction in fluorescence intensity was again observed at the highest dose (75 µg/mL).

Moreover, mitochondrial membrane potential was quantitatively analyzed by means of flow cytometry, enabling the comparison of fluorescence signal distributions between treated and untreated samples. This approach allowed the assessment of membrane potential changes at the single-cell level across the analyzed conditions. For this analysis, the fluorescent probe JC-1 was used to assess mitochondrial membrane potential. First, CCCP treatment altered the JC-1 fluorescence profile of PBMCs (Figure 3), reflecting mitochondrial membrane depolarization. Representative flow cytometry plots are shown in Figure 3A,B.

Finally, the red/green fluorescence ratio of each condition was calculated and represented after normalization of the values (Figure 4). This ratio reflects the relative accumulation of JC-1 aggregates (red fluorescence) versus monomers (green fluorescence). The results followed a similar pattern observed in the microscopy analysis. In samples that were not exposed to UV light (blue dots), no significant differences were observed between the control group and the different BRCE doses, although a reduction tendency was observed at the highest dose. In contrast, in samples exposed to UV (purple squares), a significant increase in the red/green ratio was observed when cells were pre-treated with low doses of BRCE compared with the control condition, reaching statistical significance at the 2.7 µg/mL dose of BRCE. However, as the BRCE dose increased, the ratio progressively decreased, reaching values close to zero at the highest doses, which corresponds to an almost complete loss of mitochondrial membrane potential.

### 2.3. Effect of BRCE on Caspase-3 Activation

Caspase-3, a key executioner protease in the apoptotic process, is widely recognized as a reliable marker of cells undergoing programmed cell death. Results concerning caspase-3 activity were obtained using only BRCE concentrations ranging from 1 to 8.5 µg/mL, as within these doses, the protective effect of interest had been previously demonstrated [23].

In non-irradiated samples, caspase-3 staining was absent or detected at very low levels, indicating minimal basal apoptotic activity under these conditions (Figure 5A). In contrast, UV-exposed samples showed a marked increase in caspase-3 activation, with strong fluorescence observed in the untreated control group, confirming the induction of apoptosis (Figure 5B). Notably, treatment with BRCE led to a clear reduction in fluorescence intensity compared to the UV-treated control without BRCE (Figure 5C). Notably, although only the most representative images are presented, treatment with 1 µg/mL BR showed a pattern of caspase-3 staining comparable to that observed at 2.7 µg/mL. However, this effect was no longer evident at 8.5 µg/mL, where the caspase-3 staining was comparable to that of the untreated group.

### 2.4. Effect of BRCE on Survival- and Immune-Related Markers: Bcl-2 and Granzyme A

Bcl-2 expression was also evaluated under basal conditions and after UV exposure. In the absence of UV irradiation, Bcl-2 levels showed a progressive increase across increasing concentrations of BRCE, reaching statistical significance at doses of 2.7 µg/mL and 8.5 µg/mL BRCE vs. control without BRCE. In contrast, following UV exposure, Bcl-2 expression exhibited a more pronounced increase across the tested conditions compared to the basal level, reaching greater statistical significance at the same concentrations of BRCE (Figure 6).

Granzyme A expression was also evaluated in PBMCs treated with BRCE under basal (non-irradiated) conditions and following UV-induced apoptosis (Figure 7). In non-irradiated samples, Granzyme A median fluorescence intensity (MFI) remained largely comparable to the control group across the lower and intermediate BRCE concentrations, indicating a stable basal expression. Only the highest dose tested (8.5 µg/mL) produced a slight but significant increase in Granzyme A levels. In contrast, under UV-induced conditions, BRCE treatment led to an earlier and more evident increase in Granzyme A levels, with elevations already detectable at lower concentrations compared to the basal setting, with significant differences at doses of 2.7 µg/mL and 8.5 µg/mL. This response was more pronounced as the concentration increased, showing a clearer dose-dependent trend under apoptotic stress. Although absolute MFI values were lower than those of other intracellular markers like Bcl-2, the differences between BRCE-treated and control groups were statistically significant, indicating a modulatory effect of BRCE on Granzyme A expression that becomes more evident in the presence of UV-induced stress.

## 3. Discussion

Ultraviolet-induced stress represents a major trigger of oxidative damage and apoptotic cell death in immune cells, including PBMCs, leading to impaired cellular function and viability [1]. In this context, natural carotenoids have attracted significant attention due to their ability to counteract oxidative stress and modulate apoptotic responses [24,25]. Among them, bacterioruberin has recently emerged as a promising candidate with potential cytoprotective effects [26]. As a C_50_ carotenoid, bacterioruberin belongs to a group of pigments that, as previously mentioned, have gained increasing scientific interest, with studies demonstrating a key role for C_50_ carotenoids in regulating membrane fluidity [27] and in conferring UV-photoprotection. In this regard, a novel Antarctic Arthrobacter strain has been shown to produce C_50_ carotenoids, likely decaprenoxanthin and/or sarcinaxanthin, with enhanced carotenoid accumulation correlating with increased resistance to UV-B irradiation [28]. Similarly, metabolic engineering of *Corynebacterium* *glutamicum* has enabled the overproduction of decaprenoxanthin, reported to possess UV-A protectant properties, providing the first determination of its antioxidant activity [29].

Previous studies from our group have demonstrated that BRCE improves PBMCs’ viability and reduces UV-induced apoptosis, supporting a protective role of this compound in cellular stress conditions [23]. The present study extends these findings by providing a more comprehensive evaluation of oxidative stress, mitochondrial integrity, and apoptotic regulation, including ROS production, mitochondrial membrane potential, caspase-3 activation, Bcl-2 expression, and granzyme A levels.

Our results showed that BRCE reduces intracellular ROS levels following UV exposure, indicating a decrease in oxidative stress, which is one of the earliest events in UV-induced cellular damage [30]. It should be noted that the DCFH-DA assay reflects a general reduction in intracellular oxidative activity rather than the identification of a specific ROS/RNS species or a demonstrated antioxidant/scavenging mechanism attributable to BRCE. Previous evidence also shows that a wide range of carotenoids, including lutein, zeaxanthin, β-carotene, and lycopene, effectively reduce UV-induced ROS generation and oxidative stress in both cellular and clinical models [31,32]. Specifically, in vitro studies using human dermal fibroblasts have demonstrated that carotenoids such as β-carotene and lycopene attenuate UV-induced oxidative stress and photodamage by reducing ROS formation and oxidative stress-related gene expression responses [33]. In addition, keratinocyte and epithelial cell models have shown that lutein and zeaxanthin suppress ROS production and inflammatory signaling following UV exposure, thereby improving cellular resistance to oxidative injury [34]. Complementing these in vitro findings, in vivo studies have reported that dietary supplementation with carotenoids reduces UV-induced oxidative stress, ROS, and photodermal damage in animal models and human skin, further supporting their systemic photoprotective effects [35,36,37].

Consistent with these observations, astaxanthin, one of the most extensively studied antioxidant carotenoids, has also been reported to attenuate UV-induced oxidative stress in cellular models. In normal human epidermal keratinocytes, UVB irradiation significantly increased intracellular ROS levels, whereas pretreatment with astaxanthin markedly reduced ROS production in a dose-dependent UV exposure model. These results support the ability of carotenoids to counteract ROS generation following UV-induced stress [38]. Interestingly, the antioxidant efficiency of carotenoids has been associated with structural characteristics such as the number of double conjugated bonds, the length of the conjugated polyene chain, and the presence of polar functional groups, which enhance their capacity for free-radical scavenging and singlet oxygen quenching [21]. In this context, bacterioruberin, the major carotenoid present in BRCE, exhibits distinctive structural features compared with most conventional dietary carotenoids, including an extended conjugated double-bond system (up to 13 double bonds) and four hydroxyl groups. These properties have been linked to enhanced antioxidant performance and improved structural stability and functions of the biological membranes in which this natural pigment is inserted in microbial bacterioruberin producers [15]. Accordingly, the marked reduction in intracellular ROS levels observed in UV-exposed PBMCs following BRCE treatment is compatible with these structural attributes; however, free-radical scavenging and singlet oxygen quenching were not specifically evaluated in the present study, and therefore, a direct mechanistic link between these structural properties and the observed effects cannot be established from our data alone.

However, at higher concentrations, the pronounced reduction in ROS levels may not necessarily reflect enhanced antioxidant activity. Rather, it is likely associated with BRCE-induced cytotoxicity, as previously reported [23], which may compromise cellular integrity and viability. Under these conditions, reduced metabolic activity and loss of viable cells could contribute to an apparent decrease in ROS production. Therefore, while BRCE appears to exert protective effects against UV-induced ROS at lower doses, higher concentrations might introduce toxic effects.

Moreover, a loss of mitochondrial membrane potential (ΔΨm) is also a well-established early event in UV-induced apoptosis, reflecting mitochondrial dysfunction and activation of the intrinsic apoptotic pathway. UV radiation induces mitochondrial depolarization in different cellular models, including keratinocytes, contributing to the initiation of apoptotic signaling [39]. In this context, carotenoids such as astaxanthin have been reported to exert protective effects by preserving mitochondrial integrity and attenuating UV- or oxidative stress-induced decreases in ΔΨm [40]. Similarly, other carotenoid-related antioxidants have been shown to counteract stress-induced mitochondrial dysfunction by maintaining mitochondrial membrane polarization and improving mitochondrial stability [25]. In agreement with these findings, the present study shows that BRCE, at the used doses, preserves mitochondrial membrane potential in non-UV-exposed PBMCs and seems to restore it in UV-irradiated PBMCs, suggesting a non-cytotoxic effect in the former and a stabilization of mitochondrial integrity and reduced activation of the intrinsic apoptotic pathway in the latter. These results extend previous observations to an immune cell model [23] and support the role of BRCE in protecting mitochondrial function under UV-induced stress conditions. Notably, this mitochondrial response appears to follow a biphasic pattern, with low concentrations of BRCE associated with improved mitochondrial membrane potential, whereas higher concentrations lead to a progressive loss of this protective effect. This pattern is consistent with the dose-dependent behavior observed in the ROS analysis, as well as with our previous report showing a concentration-dependent cytoprotective effect of BRCE in UVB-exposed PBMCs [23]. Taken together, these findings suggest a hormetic-like response, where low doses of BRCE may activate adaptive protective responses, while higher doses may disrupt cellular homeostasis and compromise mitochondrial function. Biphasic, hormetic-like dose responses have been increasingly documented for phytochemicals, with low concentrations exerting protective or adaptive effects and higher concentrations producing opposite or detrimental outcomes [41]. This concept has been further discussed in terms of its underlying mechanisms and limitations as a general biological principle [42] and has also been described for natural bioactive antioxidants in the context of inflammation, further supporting the biological plausibility of dose-dependent hormetic effects such as those observed in the present study [43].

Furthermore, these effects are consistent with the observed decrease in caspase-3 activation, which reflects inhibition of the downstream execution phase of apoptosis. BRCE treatment reduces caspase-3 activation in UV-irradiated PBMCs, indicating a decrease in the execution phase of apoptosis. The reduction in caspase-3 activation observed in BRCE-treated PBMCs following UV exposure agrees with previously reported effects of carotenoids on apoptotic execution pathways. Astaxanthin has been shown to attenuate UVB-induced apoptosis in human epidermal keratinocytes by reducing caspase-3 activation, thereby limiting the execution phase of programmed cell death [38]. In neuronal cell models, β-carotene has been shown to reduce the activity of caspases-3, -8 and -9, highlighting its ability to modulate both intrinsic and extrinsic apoptotic pathways [44]. In line with these findings, topical lycopene has been shown in in vivo mouse models to suppress UVB-induced apoptotic cascades, as evidenced by reduced caspase-3 expression, an effect attributed to the inhibition of UV-induced DNA damage and/or enhancement of DNA repair mechanisms, thereby preventing downstream apoptotic execution [45]. Therefore, the observed decrease in caspase-3 activation following BRCE treatment in PBMCs may be indicative of a broader inhibitory effect on upstream apoptotic signaling, contributing to the overall preservation of cell viability under UV-induced stress conditions.

Given the close relationship between oxidative stress, mitochondrial dysfunction, and apoptotic signaling, particular attention was next given to Bcl-2, a key anti-apoptotic protein involved in the regulation of mitochondrial integrity and cell survival under stress conditions. In this context, UV irradiation has been reported to downregulate Bcl-2 expression both in vitro and in vivo, thereby favoring a pro-apoptotic environment and contributing to mitochondrial destabilization and the progression of apoptosis [46].

In contrast, BRCE increased Bcl-2 levels under both basal and UV-exposed conditions, indicating an active enhancement of cell survival signaling that is not solely dependent on UV-mediated downregulation. This pattern may reflect a cell-type-dependent regulation of Bcl-2 in response to UV-induced stress. Nevertheless, astaxanthin has been shown to restore Bcl-2 expression and modulate the balance between pro- and anti-apoptotic proteins in UVB-exposed keratinocytes [38]. In agreement with the cytoprotective role of carotenoids, BRCE increased Bcl-2 expression in PBMCs under both basal and UV-induced conditions, suggesting that its effect may be independent of UV-driven downregulation and instead could reflect an active enhancement of cell survival signaling. Notably, although BRCE increased Bcl-2 levels regardless of UV exposure, slightly higher MFI values were observed in UV-irradiated cells. This observation may be explained by the activation of compensatory survival pathways following UV-induced stress, as cells can transiently upregulate anti-apoptotic mechanisms to counterbalance damage and preserve viability. Under these conditions, BRCE may further potentiate these protective responses, resulting in a greater accumulation of Bcl-2 than that observed under basal conditions. The regulation of Bcl-2 observed in the present study is consistent with its established role as a key modulator of mitochondrial apoptosis and a redox-sensitive determinant of cell fate [47]. Under UV-induced oxidative stress, cells may activate compensatory survival responses characterized by dynamic changes in Bcl-2 expression aimed at preserving mitochondrial integrity prior to the commitment to apoptosis. In this context, the effects of BRCE may be explained by its ability to attenuate oxidative stress and thereby support the maintenance of pro-survival signaling pathways upstream of caspase-3 activation, which is in agreement with studies in neuronal cell models showing that β-carotene can disrupt the apoptotic cascade by reducing caspase-3, -8 and -9 activity and by promoting a shift in the BAX/BCL-2 balance toward cell survival through upregulation of BCL-2 expression [44]. Overall, these findings support the role of BRCE as a potential cytoprotective agent that promotes anti-apoptotic signaling and helps maintain mitochondrial integrity under UV-induced stress conditions.

Finally, granzyme A was evaluated intracellularly as a marker associated with cytotoxic lymphocyte populations within the PBMC fraction, rather than as a direct effector of apoptosis in the total cell population. In this context, its expression reflects the presence and functional state of NK cells and cytotoxic T lymphocytes, which constitute a variable subset of PBMCs, rather than apoptotic activity [48]. Given that granzyme A is primarily associated with the cytotoxic function of lymphocytes rather than intrinsic apoptotic signaling, its relatively stable levels shown in this study under basal and UV-exposed control conditions may reflect the absence of a strong immune activation stimulus induced by UV irradiation under the experimental conditions used. Moreover, this modest response is also consistent with the fact that the granzyme A-mediated cytotoxic pathway is largely caspase-independent [49,50,51], which may further explain the limited magnitude of changes observed at the total PBMC level.

In contrast, BRCE treatment modulated granzyme A expression in a dose- and condition-dependent manner. While effects under basal conditions were limited and mainly observed at higher concentrations, UV-exposed cells exhibited a more pronounced and earlier response, suggesting that BRCE influences cytotoxic lymphocyte activity more strongly under stress conditions. This pattern is consistent with previous reports showing that carotenoids can enhance cytotoxic lymphocyte function, including NK and CD8^+^ T cell activity, which is closely associated with granzyme-mediated cytotoxicity [52]. In addition, astaxanthin has been reported to increase granzyme A expression in NK cells and CD8^+^ T lymphocytes [53], supporting the immunomodulatory potential of carotenoids in cytotoxic immune compartments.

This response may be further influenced by the ability of immune cells to adapt to oxidative stress conditions, in which modulation of cytotoxic effector molecules can occur as part of a broader stress-response program. The more pronounced changes observed following UV exposure may therefore reflect the activation of adaptive stress-response behavior within cytotoxic lymphocyte populations, with BRCE contributing to the preservation of their functional competence. Importantly, these changes occurred alongside the previously observed improvements in cell viability, suggesting maintenance of immune effector capacity rather than enhanced cell death, thereby further supporting the overall cytoprotective profile of BRCE.

Importantly, the interpretation of granzyme A must be considered independently from caspase-dependent apoptotic markers such as caspase-3 and Bcl-2. While caspase-3 reflects the execution phase of mitochondrial apoptosis at the total cell population level, granzyme A reflects the functional status of cytotoxic lymphocytes. Accordingly, BRCE appears to exert a dual protective role, enhancing cell survival signaling while simultaneously modulating granzyme A expression patterns under UV-induced oxidative stress.

Taken together, the present findings identify BRCE as a multifunctional cytoprotective agent that integrates antioxidant and anti-apoptotic effects with modulatory effects on immune cell markers under UV-induced stress conditions in PBMCs. Specifically, BRCE attenuates oxidative stress, preserves mitochondrial membrane potential, and inhibits apoptotic execution through caspase-3 downregulation and Bcl-2 upregulation, while also contributing to the maintenance of cytotoxic immune cell functionality as indicated by granzyme A modulation. Collectively, these results highlight BRCE as a promising bioactive compound capable of enhancing cellular resilience to UV-induced stress while preserving immune cell viability and function. The limited availability of studies evaluating carotenoids in UV-exposed in vitro models restricts direct comparison with previous findings, whereas bacterioruberin, a C50 carotenoid, represents an increasingly relevant class of natural compounds in the context of oxidative stress biology; in this context, the present work provides one of the first experimental frameworks describing its cytoprotective effects under UV-induced stress. Although BR is known to insert into and stabilize lipid membranes owing to its elongated polyene structure, whether it may also exert a direct extracellular effect contributing to the observed cytoprotection cannot be ruled out. Overall, these findings support the development of BRCE as a UV-protective strategy with potential applications in photoprotection, nutraceutical formulations, and adjunct approaches targeting oxidative stress-related immune dysfunction.

## 4. Materials and Methods

### 4.1. Reagents

RPMI 1640 medium (Biowest, Nuaillé, France) was used as the basal culture medium and supplemented with 10% fetal bovine serum (FBS; Gibco–Thermo Fisher Scientific, Waltham, MA, USA), 2 mM L-glutamine, and antibiotics consisting of 100 U/mL penicillin and 100 μg/mL streptomycin (Biowest, Nuaillé, France). Phosphate-buffered saline (PBS; HyClone, Logan, UT, USA) was also used in the study as a washing buffer.

The reagents employed included 2′,7′-dichlorodihydrofluorescein diacetate (DCFH-DA; Cayman Chemical, Ann Arbor, MI, USA), carbonyl cyanide 3-chlorophenylhydrazone (CCCP; Sigma-Aldrich, St. Louis, MO, USA), JC-1 (5,5′,6,6′-tetrachloro-1,1′,3,3′-tetraethylbenzimidazolylcarbocyanine iodide) (APExBIO Technology LLC, Houston, TX, USA), rhodamine 123 (Sigma-Aldrich, St. Louis, MO, USA), anti-human-caspase-3 antibody (Sigma-Aldrich, St. Louis, MO, USA), anti-human-Bcl-2-FITC antibody (BioLegend, San Diego, CA, USA), anti-human-Granzyme A-APC antibody (BioLegend, San Diego, CA, USA), Lymphocyte Separation Medium (Cultek, Madrid, Spain), paraformaldehyde (PFA) (Sigma-Aldrich, St. Louis, MO, USA), bovine serum albumin (BSA) (Sigma-Aldrich, St. Louis, MO, USA), BD Cytofix/Cytoperm™ kit (BD Biosciences, Fisher Scientific, Milford, MA, USA), Triton X-100 (Sigma-Aldrich, St. Louis, MO, USA), and 4′,6-Diamidino-2-phenylindole dihydrochloride (DAPI; Sigma-Aldrich, St. Louis, MO, USA).

### 4.2. Extraction and Quantification of Bacterioruberin-Rich Carotenoid Extract (BRCE)

BRCE extraction, purification, and purity determination were carried out following previously described protocols [16,18,54,55] using the haloarchaeal strain *Haloferax mediterranei* R-4 (ATCC 33500), originally isolated from the salt flats of Santa Pola (Alicante, Spain).

For carotenoid extraction, *H. mediterranei* cell pellets were resuspended in acetone and incubated overnight at 4 °C under continuous agitation. After incubation, the solvent was removed via filtration and the extraction process was repeated until the biomass appeared whitish, indicating effective pigment depletion. The concentration of the resulting acetone extract was determined spectrophotometrically using an Agilent Cary 60 UV–Vis spectrophotometer (Agilent Technologies, Santa Clara, CA, USA). For this purpose, 150 µL of extract was diluted in 1350 µL of acetone, and absorbance was measured at 494 nm. BRCE concentration was then calculated using the following equation [54]:

$$\left[BRCE\right]=Abs494*3.9*Diluent\;Factor$$

Subsequently, acetone was removed using a rotary evaporator (Rotavapor RV 3V, IKA, Staufen, Germany), and the remaining biomass was resuspended in RPMI 1640 medium via sonication to obtain a final concentration of 150 µg/mL.

The extraction procedure and the characteristic carotenoid profile of the resulting BRCE were previously established by means of HPLC and triple quadrupole liquid chromatography–mass spectrometry (LC–MS/MS) [18]. This initial analytical characterization identified BR as the predominant carotenoid, accounting for a relative abundance of 75.5 ± 1.9%, together with minor carotenoids and carotenoid precursors. Representative LC–MS/MS data from this characterization are provided in Figure S1. Subsequent BRCE preparations used in the present study were obtained following the same standardized extraction procedure. All batches were stored at 4 °C and used within one week to preserve stability.

### 4.3. Isolation of Peripheral Blood Mononuclear Cells (PBMCs)

Blood samples used in this study were obtained from 36 healthy adult volunteers through the Health Prevention and Promotion Service of the University of Alicante. Donors were allocated as follows: 8 donors for the ROS assay, 5 donors for the analysis of mitochondrial membrane potential via fluorescence microscopy, 8 donors for the analysis of mitochondrial membrane potential via flow cytometry, 5 donors for caspase-3 analysis (although representative confocal images from only one donor are shown, as consistent results were observed across all donors), 5 donors for Bcl-2 analysis, and 5 donors for Granzyme A analysis. All procedures were conducted in accordance with the ethical standards of the institutional research committee and with the principles of the Declaration of Helsinki. The study protocol was reviewed and approved by the Ethics Committee of the University of Alicante (Expedient UA-2025-05-28_1), and written informed consent was obtained from all participants prior to blood collection.

Briefly, blood samples were collected in EDTA tubes, and PBMC isolation was performed using a density gradient separation technique. Whole blood was first diluted 1:1 with PBS 1X and carefully layered over lymphocyte separation medium in a 2:1 ratio. Samples were centrifuged at 360× *g* for 30 min at room temperature. The PBMC layer was then collected and washed twice with 10 mL of PBS 1X to remove residual plasma and platelets. Then, they were resuspended in complete RPMI medium, reaching a final concentration of 1 × 10^6^ cells/mL.

### 4.4. PBMC Culture Preparation, BRCE Treatment, and UV Irradiation

After resuspension, cells were seeded into 96-well plates at a density of 100,000 cells per well in a final volume of 200 µL, with six different final concentrations of BRCE (0, 1, 2.7, 8.5, 25, and 75 µg/mL), a range chosen according to our previous studies [19,23]. Then, PBMCs were incubated for 24 h at 37 °C and 5% CO_2_. Finally, cells were treated with UV-B light (312 nm) using a transilluminator (TFX-20M, Life Technologies, Carlsbad, CA, USA) with 9 mW/cm^2^ intensity for 2 min and incubated again for another 2 h (for ROS analysis) or 24 h (for mitochondrial membrane potential, caspase 3, and apoptosis-related protein analysis) at 37 °C and 5% CO_2_. The different incubation times were also previously determined through optimization experiments [23]. The negative control consisted of PBMCs unexposed to UV radiation, whereas the positive control comprised UV-exposed PBMCs in the absence of BRCE. Under these conditions, the expected cellular response was observed, confirming that the experimental system reliably detects UV-induced cellular damage.

### 4.5. Intracellular Reactive Oxygen Species (ROS) Measurement

ROS levels were assessed using the fluorescent probe DCFH-DA, a cell-permeable compound that is deacetylated by intracellular esterases and subsequently oxidized by ROS to generate the highly fluorescent product 2′,7′-dichlorofluorescein (DCF). This probe is particularly sensitive to intracellular hydrogen peroxide (H_2_O_2_), hydroxyl radicals (•OH), and peroxynitrite. Therefore, this assay provides a general assessment of intracellular oxidative activity rather than the identification of a specific ROS species.

Following UV exposure and a 2 h incubation period, PBMCs were washed and incubated with 10 µM DCFH-DA for 30 min at 37 °C in the dark. After incubation, cells were washed with PBS 1X to remove excess probe, and fluorescence intensity was measured via flow cytometry (Northern Lights, Cytek, Fremont, CA, USA).

### 4.6. Mitochondrial Membrane Potential (Δψm) Analysis

Δψm was assessed using the fluorescent probe JC-1, a cationic dye that accumulates in mitochondria in a potential-dependent manner, forming red-fluorescent aggregates in cells with high Δψm, whereas it remains as green-fluorescent monomers in depolarized or apoptotic cells.

Following incubation of PBMCs with the indicated concentrations of BRCE and subsequent UV exposure, cells were washed with PBS 1X and stained with 2 µM JC-1 for 30 min at 37 °C in the dark. Cells were then washed again and immediately analyzed via flow cytometry. As a positive control for mitochondrial depolarization, cells were pretreated with 50 µM carbonyl cyanide 3-chlorophenylhydrazone (CCCP) for 10 min at 37 °C, a condition known to collapse the mitochondrial proton gradient and induce loss of Δψm, resulting in predominant green fluorescence.

Mitochondrial function was further evaluated using Rhodamine 123, a cationic fluorescent dye that accumulates in mitochondria in proportion to Δψm. After UV exposure, PBMCs were washed with PBS 1X and incubated with rhodamine 123 for 10 min at 37 °C in the dark. Cells were then washed twice to remove excess dye and immediately visualized using a fluorescence microscope (Leica DM750, Wetzlar, Germany). Fluorescence images were acquired under identical exposure and acquisition settings, and mean cellular fluorescence intensity was quantified by measuring gray values at the single-cell level.

### 4.7. Caspase-3 Immunofluorescence Analysis

Caspase-3 activation was assessed by means of immunofluorescence staining followed by confocal laser scanning microscopy analysis. Briefly, cells were fixed with 4% paraformaldehyde and permeabilized with 1% Triton to allow intracellular access of antibodies. After blocking non-specific binding sites, samples were incubated for 1 h at 37 °C with a 1/100 primary antibody against active caspase-3, followed by a 37 °C and 45’ incubation with an appropriate fluorophore-conjugated secondary antibody with Alexa Fluor 568 at 1/500. Cell nuclei were counterstained with DAPI. Samples were then mounted on glass slides and visualized using a confocal laser scanning microscope (Zeiss LSM-800). Images were acquired under identical acquisition settings across all experimental conditions to ensure comparability of fluorescence intensity and localization.

### 4.8. Intracellular Bcl-2 and Granzyme A Expression Analysis

Analysis of apoptosis-related proteins (Bcl-2 and Granzyme A) was performed by means of intracellular immunofluorescence staining followed by flow cytometry. Briefly, following UV exposure and the corresponding incubation period, cells were washed, fixed and permeabilized using a commercial fixation and permeabilization kit (BD Cytofix/Cytoperm™) according to the manufacturer’s instructions to allow intracellular access of antibodies. After blocking non-specific binding sites, samples were incubated for 30 min with fluorochrome-conjugated antibodies against Bcl-2 (Bcl-2-FITC) and Granzyme A (Granzyme A-APC). Following incubation, cells were washed to remove unbound antibodies and immediately analyzed via flow cytometry. Fluorescence signals corresponding to FITC and APC channels were recorded to quantify Bcl-2 and Granzyme A expression, respectively, and data were analyzed across the different experimental conditions.

### 4.9. Data Analysis

Flow Cytometry analysis was performed with the SpectroFlo software 4.0.4. (Cytek, Fremont, CA , USA). Fluorescence microscopy images were measured using ImageJ 1.54g software (National Institutes of Health, Bethesda, MD, USA). Confocal microscopy images were obtained and analyzed using ZEN Blue 3.5 (Carl Zeiss Microscopy, Jena, Germany). Descriptive statistics and statistical inference were carried out using GraphPad Prism 8 software (GraphPad Software Inc., San Diego, CA, USA). After confirming normality with the Shapiro–Wilk test, data were analysed using a paired one-way analysis of variance (ANOVA), followed by Fisher’s least significant difference (LSD) test. Statistical significance was considered at a *p*-value < 0.05.

## 5. Conclusions

This study suggests a coordinated cytoprotective effect of bacterioruberin-rich carotenoid extract (BRCE) against UV-induced stress in human peripheral blood mononuclear cells. To the best of our knowledge, this is the first study to comprehensively characterize the underlying cellular responses associated with the protective effect of BRCE, involving attenuation of oxidative stress, preservation of mitochondrial membrane potential, and modulation of key apoptosis-related and immune-associated markers, including caspase-3, Bcl-2, and granzyme A. These effects were more pronounced at low BRCE concentrations, suggesting a dose-dependent response. Overall, these findings provide mechanistic insight into the bioactivity of bacterioruberin and reinforce its relevance as an emerging C_50_ carotenoid with strong photoprotective and immunomodulatory potential and notable modulatory effects on immune cells. This supports its further exploration for potential applications in preventing oxidative stress-related cellular damage, as well as in nutraceutical, cosmetic, and adjunct therapeutic strategies.

## Figures and Tables

**Figure 1:**
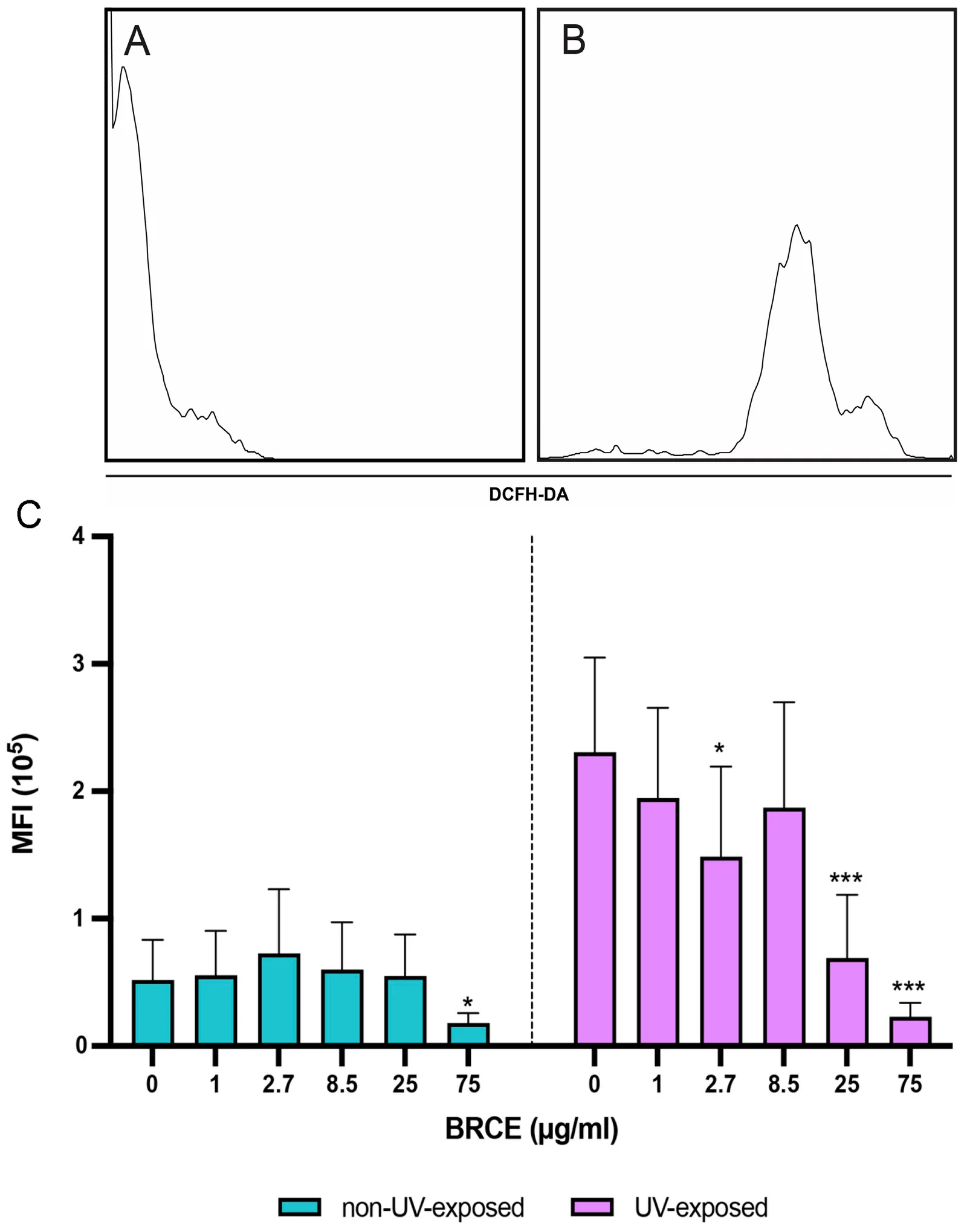
Analysis of intracellular reactive oxygen species (ROS) production in peripheral blood mononuclear cells (PBMCs). Representative histograms showing (**A**) Basal PBMCs (negative control) and (**B**) PBMCs following ultraviolet (UV) irradiation (2 min) and subsequent incubation for 2 h. (**C**) Quantification of intracellular ROS levels in PBMCs after 24 h of pre-incubation with the different concentrations of bacterioruberin-rich carotenoid extract (BRCE) followed by a 2 h UV incubation period. Blue bars represent non-UV-exposed samples, whereas purple bars represent UV-exposed samples (*n* = 8). Data are presented as mean ± standard deviation (SD). Statistical significance was determined via one-way analysis of variance (ANOVA) followed by Fisher’s least significant difference (LSD) post hoc test. * *p* < 0.05; *** *p* < 0.001 vs. controls without BRCE.

**Figure 2:**
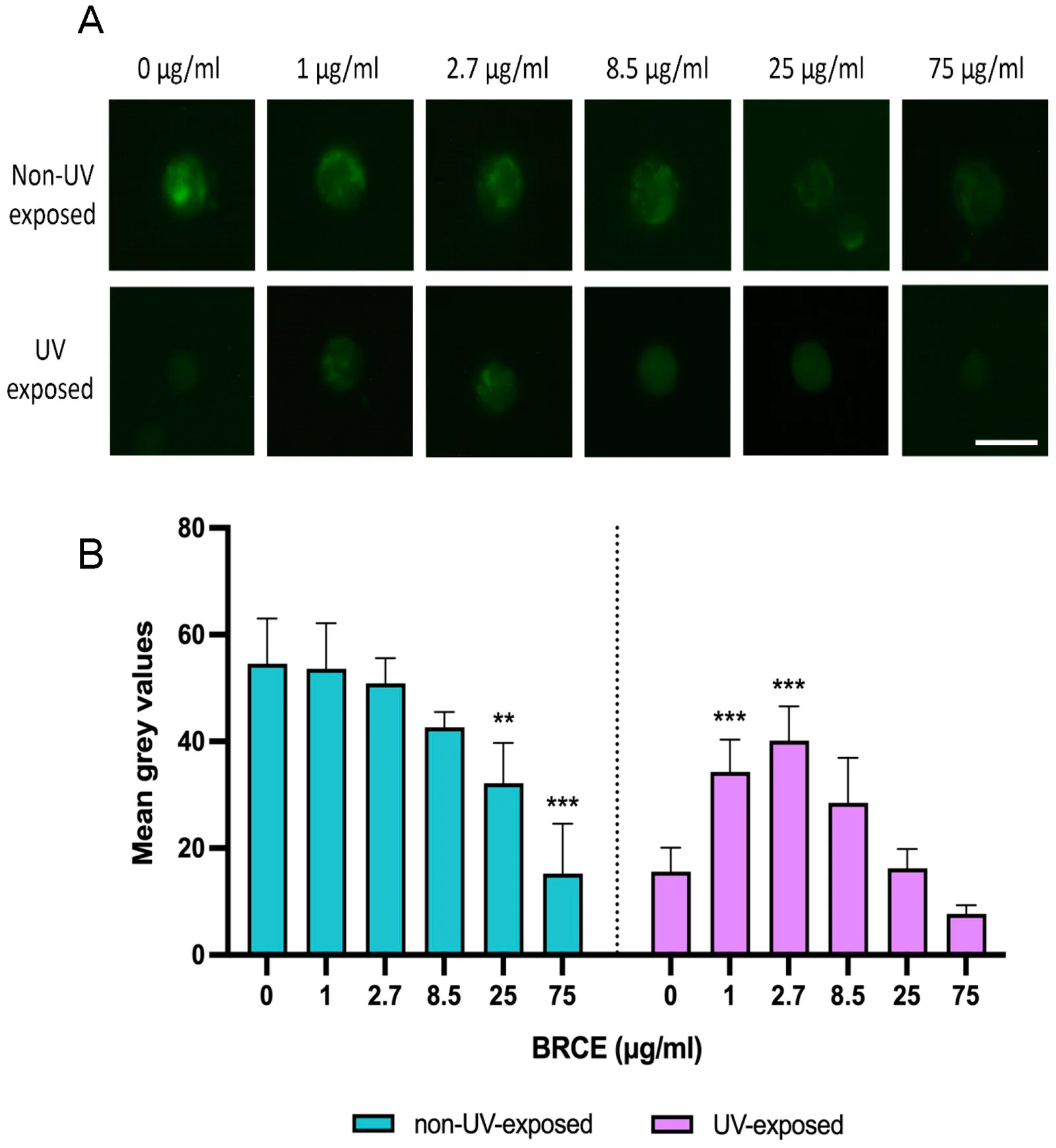
Analysis of mitochondrial membrane potential (Δψm) in PBMCs (**A**) Fluorescence microscopy images and quantification of mean gray values of cells treated with increasing concentrations of bacterioruberin-rich carotenoid extract (BRCE). (**B**) Bar graph showing mean grey values under both conditions (without UV exposure, blue bars; after UV exposure, red bars) (*n* = 5). Data are presented as mean ± standard deviation (SD). Statistical significance was assessed using one-way analysis of variance (ANOVA) followed by Fisher’s least significant difference (LSD) test. ** *p* < 0.01; *** *p* < 0.001 vs. controls without BRCE. Scale bar = 5 µm.

**Figure 3:**
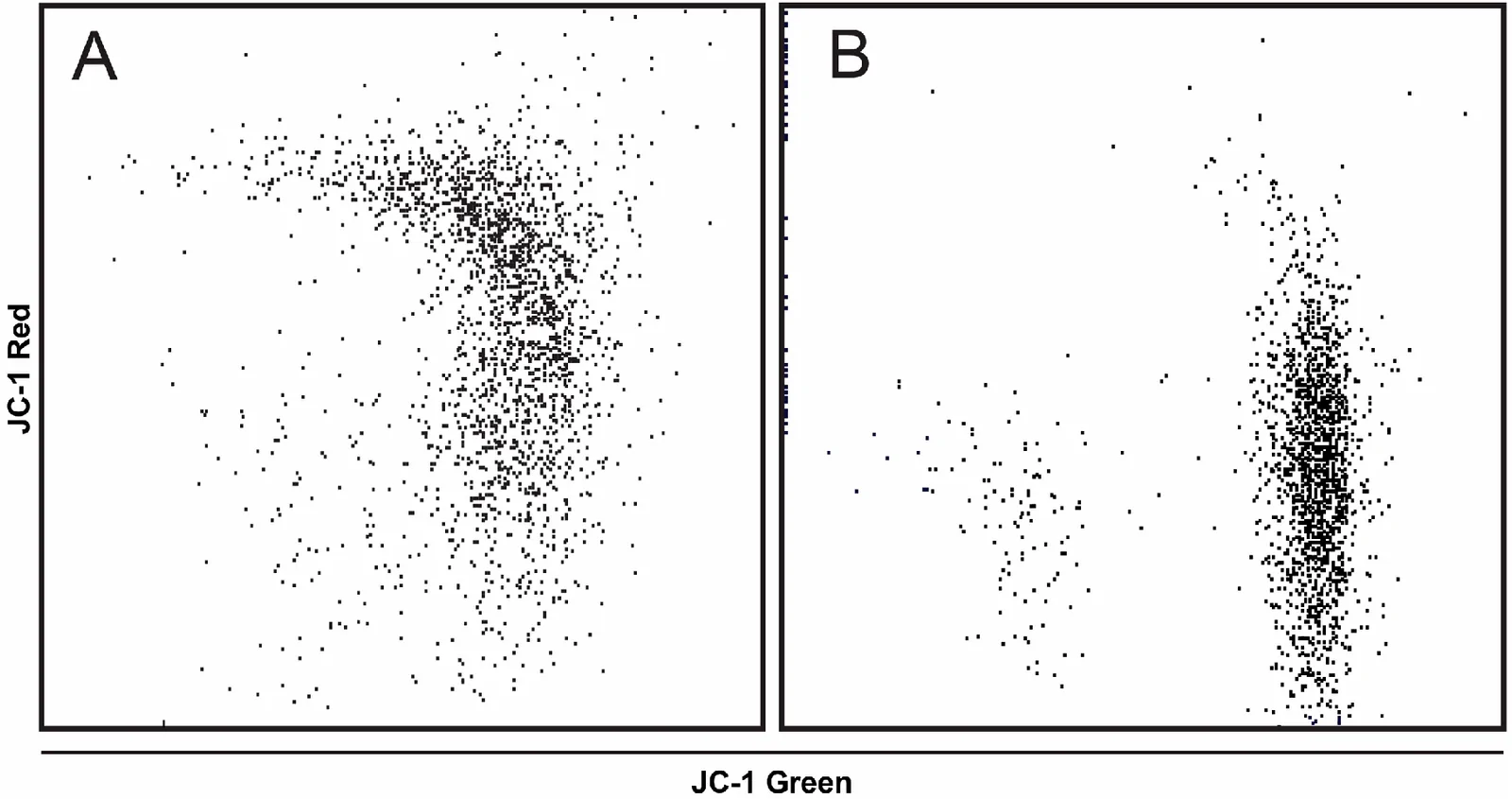
Representative flow cytometry analysis from one donor of JC-1 staining. (**A**) PBMCs stained with JC-1; (**B**) JC-1-stained PBMCs pretreated with CCCP.

**Figure 4:**
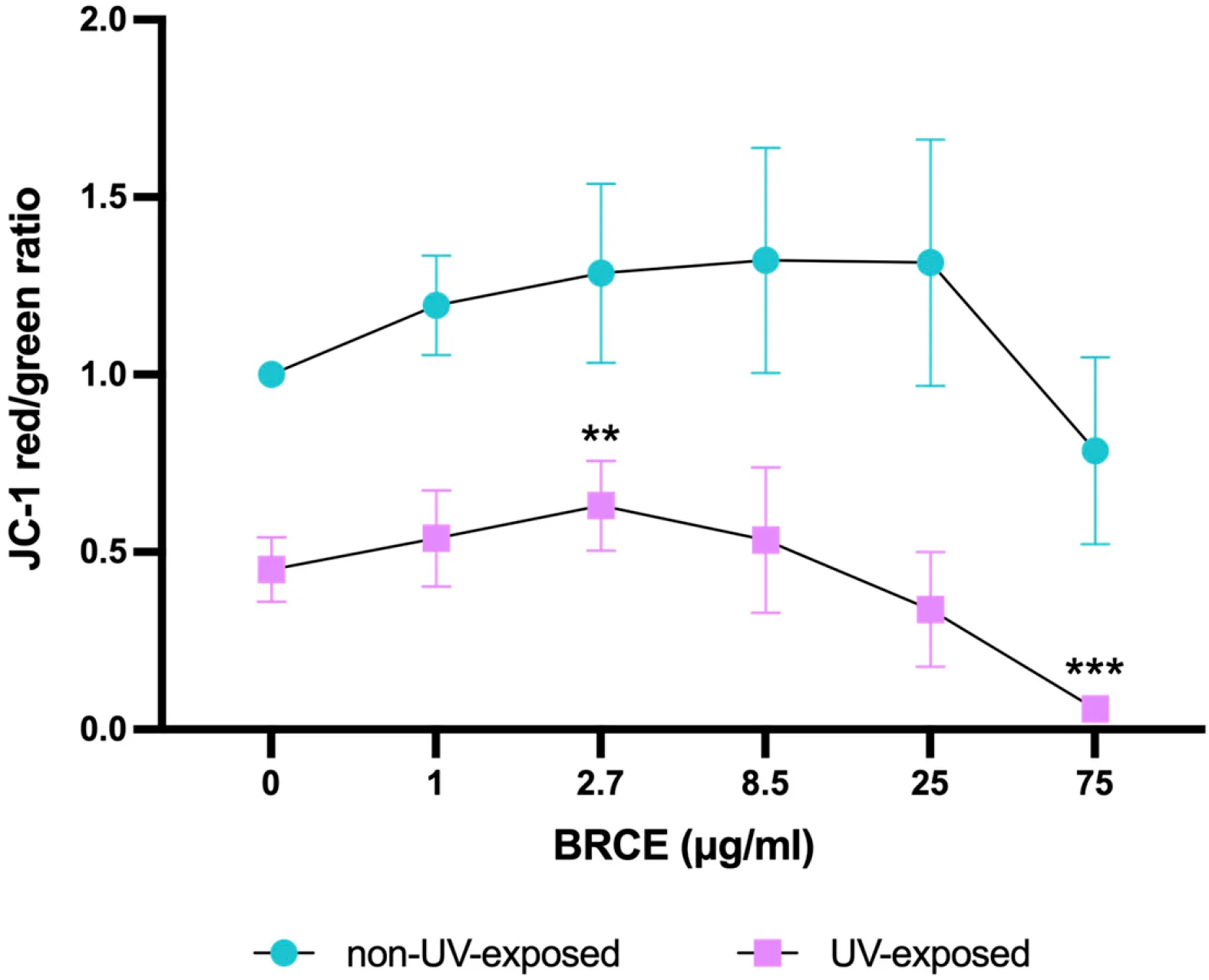
Analysis of mitochondrial membrane potential using the JC-1 probe. Mitochondrial membrane potential was assessed based on the ratio of red fluorescence (JC-1 aggregates) to green fluorescence (JC-1 monomers) and analyzed by means of flow cytometry (*n* = 8). Graph showing normalized values of cells pre-incubated with increasing concentrations of BRCE under two conditions: without UV exposure (blue dots) and with UV exposure (purple squares). Data represent mean ± SD. Statistical significance was determined using analysis of variance (ANOVA) followed by Fisher’s least significant difference (LSD) test. ** *p* < 0.01; *** *p* < 0.001 vs. controls without BRCE.

**Figure 5:**
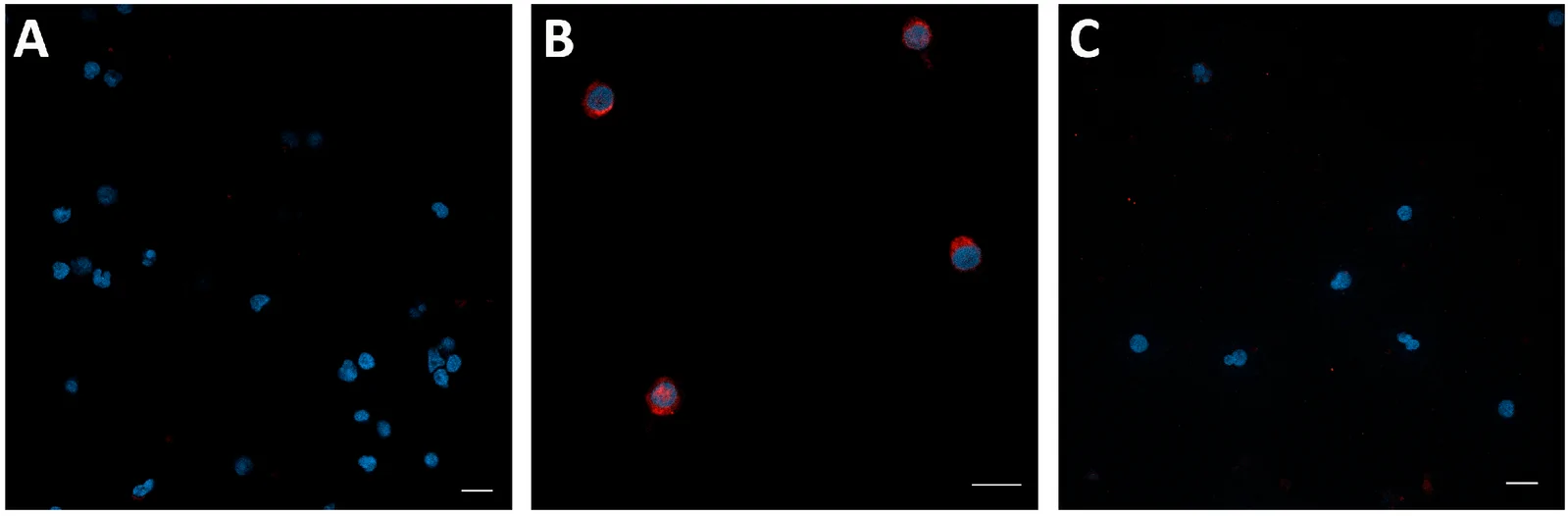
Confocal microscopy images of caspase-3 expression in PBMCs (representative images from one donor). (**A**) Non-UV-exposed control cells. (**B**) PBMCs exposed to UV irradiation without BRCE treatment. (**C**) PBMCs exposed to UV irradiation and pretreated with 2.7 µg/mL BRCE. Nuclei are shown in blue (DAPI) and caspase-3 in red (AF568) in the merged images. Scale bar = 10 µm.

**Figure 6:**
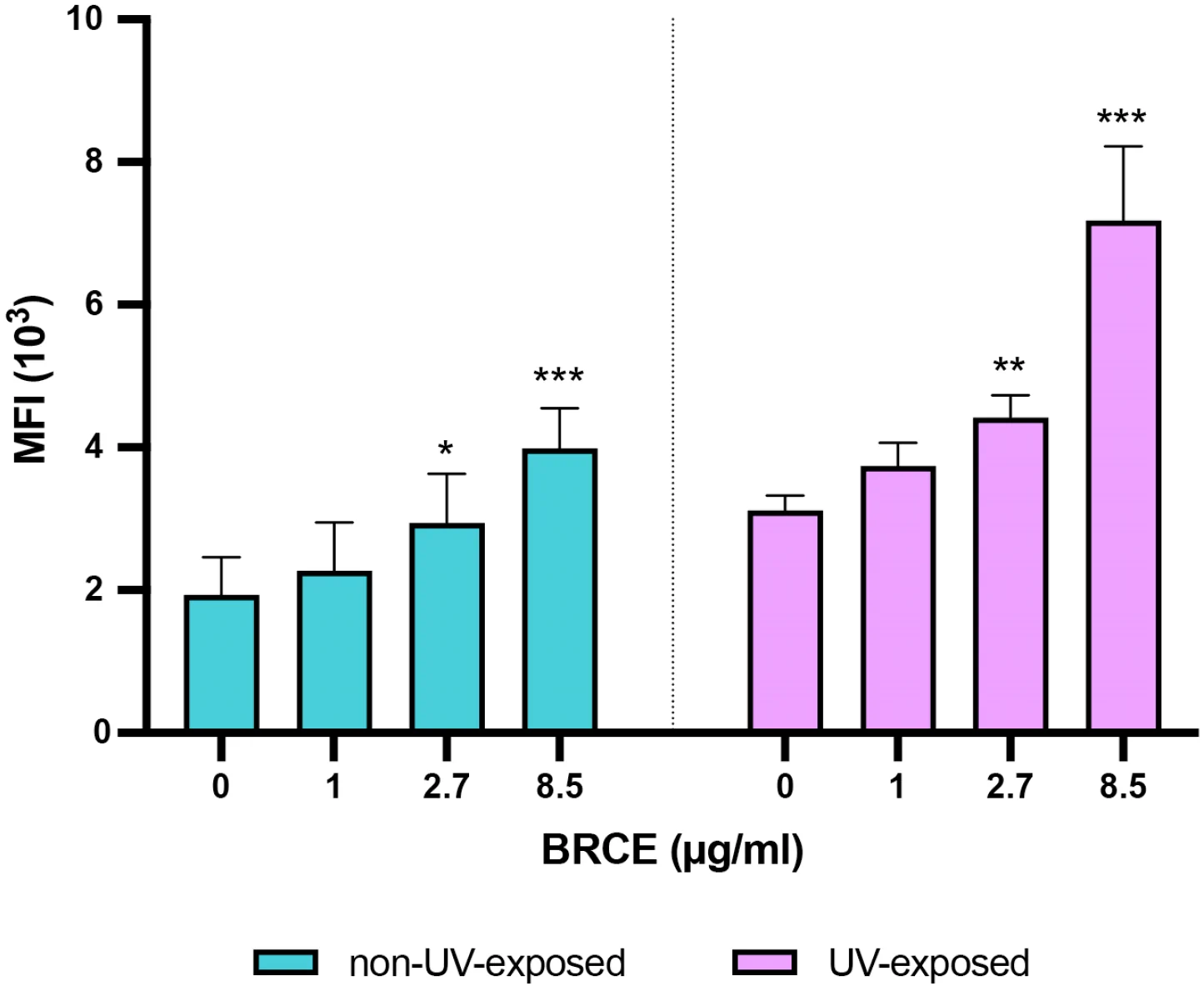
Bar graph showing Bcl-2 levels in non-irradiated (blue bars) and UV-irradiated cells (purple bars) treated with increasing concentrations of BRCE vs. control without BRCE (*n* = 5). Data represent mean ± SD. Statistical significance was determined using analysis of variance (ANOVA) followed by Fisher’s least significant difference (LSD) test. * *p* < 0.05; ** *p* < 0.01; *** *p* < 0.001 vs. controls without BRCE.

**Figure 7:**
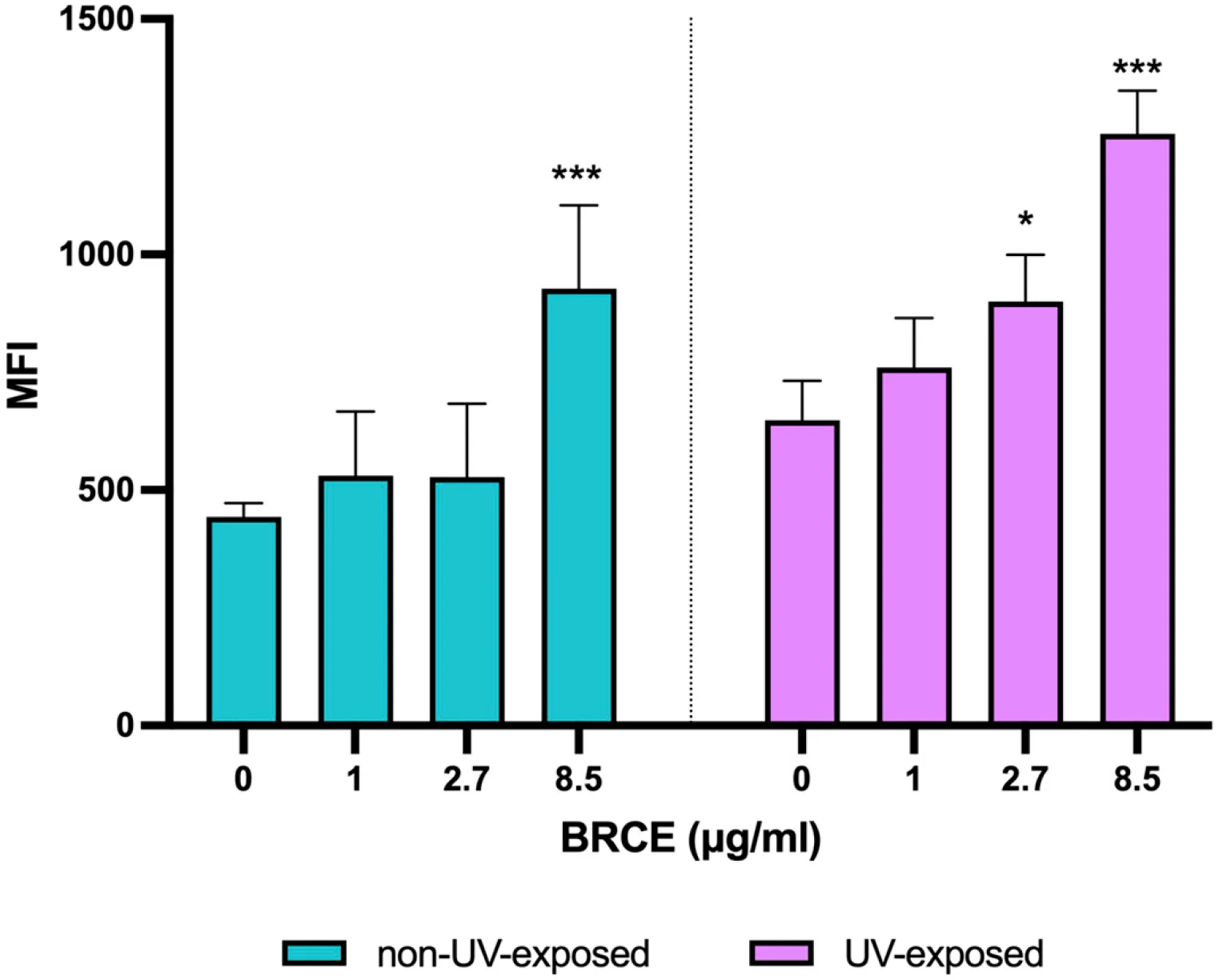
Bar graph showing Granzyme A levels in non-irradiated (blue bars) and UV-irradiated cells (purple bars) treated with increasing concentrations of BRCE vs. samples without it (*n* = 5). Data represent mean ± SD. Statistical significance was determined using analysis of variance (ANOVA) followed by Fisher’s least significant difference (LSD) test. * *p* < 0.05; ***p < 0.001 vs. controls without BRCE.

## Data Availability

All data supporting the findings of this study are available within the paper. Also, Raw FC data presented in this study have been deposited in the Repository of the University of Alicante (RUA) with URL: (https://hdl.handle.net/10045/169170; accesed on 15 July 2026). The data from the analysis with GraphPad Prism 10 are available upon request.

## Supplementary Materials

The following supporting information can be downloaded at: https://www.mdpi.com/article/10.3390/md24100335/s1, Figure S1. Representative LC–MS/MS characterization of the bacterioruberin-rich carotenoid extract (BRCE) obtained from *Haloferax (H.) mediterranei* under the culture conditions used for BRCE production. (A) Total ion chromatogram. (B) Mass spectrum obtained over the retention-time interval of 3.363–3.519 min. The predominant signal at *m*/*z* 740.6 was assigned to BR. The analytical procedure and compositional results were previously reported by Giani et al. [18].

## Abbreviations

The following abbreviations are used in this manuscript:

ANOVA	Analysis Of Variance
BCL-2	B Cell Lymphoma 2
BR	Bacterioruberin
BRCE	Bacterioruberin-Rich Carotenoid Extract
BSA	Bovine Albumin Serum
CCCP	Carbonyl Cyanide 3-Chlorophenylhydrazone
DAPI	4′,6-Diamidino-2-Phenylindole Dihydrochloride
DCF	2′,7′-Dichlorofluorescein
DCFH-DA	2′,7′-Dichlorodihydrofluorescein Diacetate
FBS	Fetal Bovine Serum
H_2_O_2_	Hydrogen Peroxide
JC-1	5,5′,6,6′-Tetrachloro-1,1′,3,3′-Tetraethylbenzimidazolylcarbocyanine Iodide
LSD	Least Significant Difference
MFI	Median Fluorescence Intensity
PBMCS	Peripheral Mononuclear Cells
PBS	Phosphate-Buffered Saline
PFA	Paraformaldehyde
ROI	Region Of Interest
ROS	Reactive Oxygen Species
SD	Standard Deviation
UV	Ultraviolet
Δψm	Mitochondrial Membrane Potential
•OH	Hydroxyl Radicals

## References

[1] Hart P.H., Gorman S., Finlay-Jones J.J. (2011). Modulation of the Immune System by UV Radiation: More than Just the Effects of Vitamin D?. Nat. Rev. Immunol..

[2] Ghajar-Rahimi G., Yusuf N., Xu H. (2025). Ultraviolet Radiation-Induced Tolerogenic Dendritic Cells in Skin: Insights and Mechanisms. Cells.

[3] Salminen A., Kaarniranta K., Kauppinen A. (2022). Photoaging: UV Radiation-Induced Inflammation and Immunosuppression Accelerate the Aging Process in the Skin. Inflamm. Res..

[4] Paz M.L., Maglio D.H.G., Weill F.S., Bustamante J., Leoni J. (2008). Mitochondrial Dysfunction and Cellular Stress Progression after Ultraviolet B Irradiation in Human Keratinocytes. Photodermatol. Photoimmunol. Photomed..

[5] Xie Y., Chen Z., Wu Z. (2022). Four-Octyl Itaconate Attenuates UVB-Induced Melanocytes and Keratinocytes Apoptosis by Nrf2 Activation-Dependent ROS Inhibition. Oxid. Med. Cell. Longev..

[6] Kulms D., Zeise E., Pöppelmann B., Schwarz T. (2002). DNA Damage, Death Receptor Activation and Reactive Oxygen Species Contribute to Ultraviolet Radiation-Induced Apoptosis in an Essential and Independent Way. Oncogene.

[7] Baek J.Y., Park S., Park J., Jang J.Y., Wang S.B., Kim S.R., Woo H.A., Lim K.M., Chang T.S. (2017). Protective Role of Mitochondrial Peroxiredoxin III against UVB-Induced Apoptosis of Epidermal Keratinocytes. J. Investig. Dermatol..

[8] Huang K.F., Ma K.H., Jhap T.Y., Liu P.S., Chueh S.H. (2019). Ultraviolet B Irradiation Induced Nrf2 Degradation Occurs via Activation of TRPV1 Channels in Human Dermal Fibroblasts. Free Radic. Biol. Med..

[9] Sandmann G. (2019). Antioxidant Protection from UV-and Light-Stress Related to Carotenoid Structures. Antioxidants.

[10] Medina-García M., Baeza-Morales A., Martínez-Peinado P., Pascual-García S., Pujalte-Satorre C., Martínez-Espinosa R.M., Sempere-Ortells J.M. (2025). Carotenoids and Their Interaction with the Immune System. Antioxidants.

[11] Reis-Mansur M.C.P.P., Cardoso-Rurr J.S., Silva J.V.M.A., de Souza G.R., Cardoso V.d.S., Mansoldo F.R.P., Pinheiro Y., Schultz J., Lopez Balottin L.B., da Silva A.J.R. (2019). Carotenoids from UV-Resistant Antarctic *Microbacterium* sp. LEMMJ01. Sci. Rep..

[12] Baswan S.M., Marini A., Klosner A.E., Jaenicke T., Leverett J., Murray M., Gellenbeck K.W., Krutmann J. (2020). Orally Administered Mixed Carotenoids Protect Human Skin against Ultraviolet A-Induced Skin Pigmentation: A Double-Blind, Placebo-Controlled, Randomized Clinical Trial. Photodermatol. Photoimmunol. Photomed..

[13] Hwang C.Y., Cho E.S., Kim S., Kim K., Seo M.J. (2024). Optimization of Bacterioruberin Production from *Halorubrum ruber* and Assessment of Its Antioxidant Potential. Microb. Cell Fact..

[14] Rodrigo-Baños M., Garbayo I., Vílchez C., Bonete M.J., Martínez-Espinosa R.M. (2015). Carotenoids from Haloarchaea and Their Potential in Biotechnology. Mar. Drugs.

[15] Saito T., Miyabe Y., Ide H., Yamamoto O. (1997). Hydroxyl Radical Scavenging Ability of Bacterioruberin. Radiat. Phys. Chem..

[16] Giani M., Martínez-Espinosa R.M. (2020). Carotenoids as a Protection Mechanism against Oxidative Stress in *Haloferax mediterranei*. Antioxidants.

[17] Hou J., Cui H.L. (2018). In Vitro Antioxidant, Antihemolytic, and Anticancer Activity of the Carotenoids from Halophilic Archaea. Curr. Microbiol..

[18] Giani M., Gervasi L., Loizzo M.R., Martínez-Espinosa R.M. (2022). Carbon Source Influences Antioxidant, Antiglycemic, and Antilipidemic Activities of *Haloferax mediterranei* Carotenoid Extracts. Mar. Drugs.

[19] Giani M., Montoyo-Pujol Y.G., Peiró G., Martínez-Espinosa R.M. (2023). Haloarchaeal Carotenoids Exert an In Vitro Antiproliferative Effect on Human Breast Cancer Cell Lines. Sci. Rep..

[20] Giani M., Pire C., Martínez-Espinosa R.M. (2024). Bacterioruberin: Biosynthesis, Antioxidant Activity, and Therapeutic Applications in Cancer and Immune Pathologies. Mar. Drugs.

[21] Morilla M.J., Ghosal K., Romero E.L. (2023). More Than Pigments: The Potential of Astaxanthin and Bacterioruberin-Based Nanomedicines. Pharmaceutics.

[22] Baeza-Morales A., Pascual-García S., Martínez-Peinado P., Navarro-Sempere A., Segovia Y., Medina-García M., Pujalte-Satorre C., García M.M., Martínez-Espinosa R.M., Sempere-Ortells J.M. (2025). Bacterioruberin Extract from *Haloferax mediterranei* Induces Apoptosis and Cell Cycle Arrest in Myeloid Leukaemia Cell Lines. Sci. Rep..

[23] Medina-García M., Martínez-Peinado P., Navarro-Sempere A., Segovia Y., Pascual-García S., Baeza-Morales A., Pujalte-Satorre C., López-Jaén A.B., Giani M., García M.M. (2026). Photoprotective and Cryoprotective Effect of the Carotenoid Bacterioruberin on Peripheral Blood Mononuclear Cells. Appl. Microbiol. Biotechnol..

[24] Tan B.L., Norhaizan M.E. (2019). Carotenoids: How Effective Are They to Prevent Age-Related Diseases?. Molecules.

[25] Fiedor J., Burda K. (2014). Potential Role of Carotenoids as Antioxidants in Human Health and Disease. Nutrients.

[26] Martínez-Espinosa R.M. (2025). Bacterioruberin (C50 Carotenoid): Nutritional and Biomedical Potential of a Microbial Pigment. Nutrients.

[27] Flegler A., Lipski A. (2022). The C50 Carotenoid Bacterioruberin Regulates Membrane Fluidity in Pink-Pigmented Arthrobacter Species. Arch. Microbiol..

[28] Paredes Contreras B.V., Vermelho A.B., Casanova L., de Alencar Santos Lage C., Spindola Vilela C.L., da Silva Cardoso V., Pacheco Arge L.W., Cardoso-Rurr J.S., Correa S.S., Passos De Mansoldo F.R. (2025). Enhanced UV-B Photoprotection Activity of Carotenoids from the Novel *Arthrobacter* sp. Strain LAPM80 Isolated from King George Island, Antarctica. Heliyon.

[29] Stegelmann F., Seeger J., Peters-Wendisch P., Henke N.A., Wendisch V.F. (2026). Production of Ultraviolet A Protectant C50 Carotenoid Decaprenoxanthin by Metabolically Engineered *Corynebacterium glutamicum*. Bioresour. Technol..

[30] Afaq F., Mukhtar H. (2006). Botanical Antioxidants in the Prevention of Photocarcinogenesis and Photoaging. Exp. Dermatol..

[31] Stahl W., Sies H. (2012). Photoprotection by Dietary Carotenoids: Concept, Mechanisms, Evidence and Future Development. Mol. Nutr. Food Res..

[32] Jomova K., Valko M. (2013). Health Protective Effects of Carotenoids and Their Interactions with Other Biological Antioxidants. Eur. J. Med. Chem..

[33] Offord E.A., Gautier J.C., Avanti O., Scaletta C., Runge F., Krämer K., Applegate L.A. (2002). Photoprotective Potential of Lycopene, β-Carotene, Vitamin E, Vitamin C and Carnosic Acid in UVA-Irradiated Human Skin Fibroblasts. Free Radic. Biol. Med..

[34] Ma Y., Li C., Su W., Sun Z., Gao S., Xie W., Zhang B., Sui L. (2025). Carotenoids in Skin Photoaging: Unveiling Protective Effects, Molecular Insights, and Safety and Bioavailability Frontiers. Antioxidants.

[35] Grether-Beck S., Marini A., Jaenicke T., Stahl W., Krutmann J. (2017). Molecular Evidence That Oral Supplementation with Lycopene or Lutein Protects Human Skin against Ultraviolet Radiation: Results from a Double-Blinded, Placebo-Controlled, Crossover Study. Br. J. Dermatol..

[36] Žmitek K., Žmitek J., Rogl Butina M., Hristov H., Pogačnik T., Pravst I. (2020). Dietary Lutein Supplementation Protects against Ultraviolet-Radiation-Induced Erythema: Results of a Randomized Double-Blind Placebo-Controlled Study. J. Funct. Foods.

[37] Zhang L., Liang S., Zhang Z., Wang K., Cao J., Yao M., Qin L., Qu C., Miao J. (2023). Protective Effects of ζ-Carotene-like Compounds against Acute UVB-Induced Skin Damage. Int. J. Mol. Sci..

[38] Chung B.Y., Park S.H., Yun S.Y., Yu D.S., Lee Y.B. (2022). Astaxanthin Protects Ultraviolet B-Induced Oxidative Stress and Apoptosis in Human Keratinocytes via Intrinsic Apoptotic Pathway. Ann. Dermatol..

[39] Matsumura Y., Ananthaswamy H.N. (2004). Toxic Effects of Ultraviolet Radiation on the Skin. Toxicol. Appl. Pharmacol..

[40] Nishida Y., Nawaz A., Hecht K., Tobe K. (2022). Astaxanthin as a Novel Mitochondrial Regulator: A New Aspect of Carotenoids, beyond Antioxidants. Nutrients.

[41] Jodynis-liebert J., Kujawska M. (2020). Biphasic Dose-Response Induced by Phytochemicals: Experimental Evidence. J. Clin. Med..

[42] Bondy S.C. (2023). The Hormesis Concept: Strengths and Shortcomings. Biomolecules.

[43] Barreiro-Sisto U., Fernández-Fariña S., González-Noya A.M., Pedrido R., Maneiro M. (2024). Enemies or Allies? Hormetic and Apparent Non-Dose-Dependent Effects of Natural Bioactive Antioxidants in the Treatment of Inflammation. Int. J. Mol. Sci..

[44] Khan M.I., Jeong E.S., Tasreen G., Khan M.Z., Shin J.H., Kim J.D. (2025). The Therapeutic Potential of Beta-Carotene against Neuroinflammation and Amyloid Beta in SH-SY5Y Cells. Sci. Rep..

[45] Fazekas Z., Gao D., Saladi R.N., Lu Y., Lebwohl M., Wei H. (2003). Protective Effects of Lycopene Against Ultraviolet B-Induced Photodamage. Nutr. Cancer.

[46] Isoherranen K., Sauroja I., Jansen C., Punnonen K. (1999). UV Irradiation Induces Downregulation of Bcl-2 Expression In Vitro and In Vivo. Arch. Dermatol. Res..

[47] Cory S., Adams J.M. (2002). The BCL2 Family: Regulators of the Cellular Life-or-Death Switch. Nat. Rev. Cancer.

[48] Grossman W.J., Verbsky J.W., Tollefsen B.L., Kemper C., Atkinson J.P., Ley T.J. (2004). Differential Expression of Granzymes A and B in Human Cytotoxic Lymphocyte Subsets and T Regulatory Cells. Blood.

[49] Lieberman J. (2003). The ABCs of Granule-Mediated Cytotoxicity: New Weapons in the Arsenal. Nat. Rev. Immunol..

[50] Lieberman J. (2010). Granzyme A Activates Another Way to Die. Immunol. Rev..

[51] Martinvalet D., Dykxhoorn D.M., Ferrini R., Lieberman J. (2008). Granzyme A Cleaves a Mitochondrial Complex I Protein to Initiate Caspase-Independent Cell Death. Cell.

[52] Chew B.P., Park J.S. (2004). Carotenoid Action on the Immune Response. Proceedings of the Journal of Nutrition.

[53] Park J.S., Chyun J.H., Kim Y.K., Line L.L., Chew B.P. (2010). Astaxanthin Decreased Oxidative Stress and Inflammation and Enhanced Immune Response in Humans. Nutr. Metab..

[54] Montero-Lobato Z., Ramos-Merchante A., Fuentes J.L., Sayago A., Fernández-Recamales Á., Martínez-Espinosa R.M., Vega J.M., Vílchez C., Garbayo I. (2018). Optimization of Growth and Carotenoid Production by *Haloferax mediterranei* Using Response Surface Methodology. Mar. Drugs.

[55] Giani M., Montero-Lobato Z., Garbayo I., Vílchez C., Vega J.M., Martínez-Espinosa R.M. (2021). *Haloferax mediterranei* Cells as C50 Carotenoid Factories. Mar. Drugs.

