# Supplementary material for: Bacterioruberin-Rich Carotenoid Extract Protects Human PBMCs Against UV-Induced Oxidative Stress and Apoptosis: Insights into Mitochondrial and Cell Survival Mechanisms
Source: Mar Drugs. 2026 Sep 26;24(10):335. doi: 10.3390/md24100335 (PMC13706532; doi:10.3390/md24100335)
Supplement: Supplementary file 1 [file marinedrugs-24-00335-s001.zip › marinedrugs-4472793-supplementary.pdf]

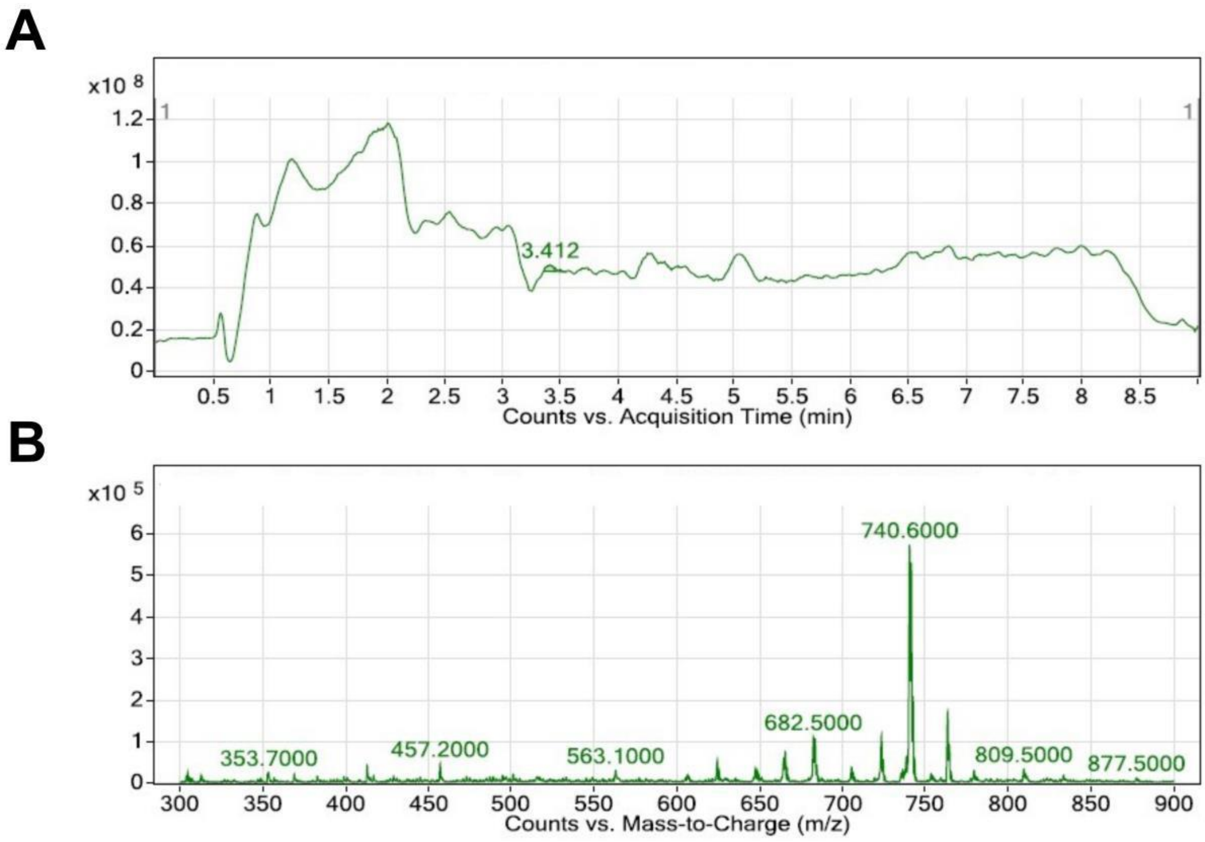

Figure S1. Representative LC–MS/MS characterization of the bacterioruberin-rich carotenoid extract (BRCE) obtained from *Haloferax (H.) mediterranei* under the culture conditions used for BRCE production. (A) Total ion chromatogram. **(B)** Mass spectrum obtained over the retention-time interval of 3.363–3.519 min. The predominant signal at m/z 740.6 was assigned to BR. The analytical procedure and compositional results were previously reported by Giani *et al.* [18].
